# First report of PCR-based detection of *Helicobacter* species DNA in *Camelus dromedarius* in Egypt

**DOI:** 10.14202/vetworld.2020.1898-1901

**Published:** 2020-09-17

**Authors:** Ahmed Youssef, Ahmed Afifi, Ayman Hamed, Mohamed Enany

**Affiliations:** 1 Department of Animal Hygiene and Zoonoses, Faculty of Veterinary Medicine, Suez Canal University, Ismailia 41522, Egypt; 2 Department of Microbiology (Bacteriology), Faculty of Veterinary Medicine, Suez Canal University, Ismailia 41522, Egypt; 3 Department of Biotechnology, Animal Health Research Institute, 7 Nady El Seid St., Dokki, Giza, Egypt

**Keywords:** *Camelus dromedarius*, *Helicobacter*, Non-*pylori*, polymerase chain reaction

## Abstract

**Background and Aim:**

*Helicobacter* species infections have epidemiological and zoonotic impacts, and different species of *Helicobacter* have been implicated in infecting humans and animals. The aim of this study was to investigate *Helicobacter* species infections in *Camelus dromedarius*.

**Materials and Methods:**

Fecal samples were collected from 32 camels from 9 camel farms located at Ismailia Governorate, Egypt. The collected samples were investigated by bacteriological isolation and conventional polymerase chain reaction (PCR) assays targeting the 16S ribosomal RNA gene.

**Results:**

Although *Helicobacter* species could not be isolated from all the examined samples, *Helicobacter* DNA was detected in 2 (22.22%) of the 9 camel farms. Of the 32 camel fecal samples examined, 4 (12.5%) were positive for *Helicobacter* species as analyzed by the PCR assay.

**Conclusion:**

To the best of our knowledge, this is the first report of PCR-based detection of *Helicobacter* species infections in *C. dromedarius*. Further epidemiological studies are required to clarify *Helicobacter* species infections in camels.

## Introduction

Following the discovery of *Helicobacter pylori* in humans, *Helicobacter* species have been reported in both wild and domestic mammals having varying dietary habits, such as dogs, cats, mice, swine, cattle, and sheep [1,2]. *Helicobacter* species that infect animals are generally termed as *Helicobacter*-like organisms, and these organisms are known to have different morphological characteristics than those of *H. pylori* (Non-*Helicobacter* *pylori* species) that commonly infect humans [3,4]. It is difficult to distinguish these non*-H. pylori* species from each other through histopathological examinations of gastric tissue sections by light microscopy [1]. Both *H. pylori* and non-*H*. *pylori* species are known to infect animals with a zoonotic mode of transmission to humans [3-5]. However, there is a lack of information regarding the role of these species in the pathogenesis of gastric diseases in animals [6].

There are various detection methods for the diagnosis of *Helicobacter* species, including invasive and non-invasive techniques. Bacteriological isolation of *Helicobacter* species encompasses several difficulties, and moreover, their isolation requires well-equipped microbiological laboratories and well-trained researchers; moreover, some *Helicobacter* species are reported to be unculturable [7]. Nonetheless, molecular detection of the DNA of *Helicobacter* spp. in clinical samples by polymerase chain reaction (PCR) assays provide a valuable tool for the identification of *Helicobacter* spp. with high sensitivity and specificity rates [7,8]. In this regard, the identification of *Helicobacter* bacteria, particularly unculturable species, using PCR assays based on the 16S ribosomal DNA gene remains the most frequently used method of detection [9].

Camels are implicated to be reservoirs of several infectious diseases, including parasitic, bacterial, and viral diseases. Some of these infectious diseases of camels are zoonotic and pose public health concerns. However, there remains a lack of information regarding *Helicobacter* infections in camels. Therefore, this study was conducted to detect *Helicobacter* spp. infections in *Camelus dromedarius*.

## Materials and Methods

### Ethical approval

This study was approved by the Research Ethics Committee of the Suez Canal University.

### Sample collection

A total of 32 fecal samples were collected from 9 camel farms located in Ismailia Governorate, Egypt, from January to March 2019. All samples were collected from apparently healthy animals. The geographic distribution of the farms was as follows: Ismailia center (n=3), Al Tal Al Kabir (n=1), Fayid (n=1), and Al Qantarah (n=4). In total, 100 g of fresh fecal sample was collected in sterile containers. The fecal specimens were transported on ice and processed within 2 h for culture to avoid reducing the viability of the organisms due to exposure to atmospheric oxygen. The samples that were not cultured on solid media within 2 h were enriched in a transport medium to maintain the viability of bacteria. Brain-heart infusion (BHI) broth (Oxoid, CM 0385B, UK) supplemented with 5% horse serum or phosphate-buffered saline at a dilution of 20% w/v was used as a transport medium to prevent dryness. The suspension of the diluted sample was sieved through a 250 μm strainer before plating onto selective media.

### Bacteriological isolation of *Helicobacter* species

One milliliter of each diluted sample was inoculated into each of two sterile test tubes containing 9 mL of BHI broth supplemented with 5% sterile horse serum. The first tube was supplemented with non-*H. pylori* selective supplement (Skirrow’s medium), whereas the second one was supplemented with *H. pylori* selective supplement (DENT, OXOID, code SR0147, UK). The inoculated test tubes were incubated at 37°C under microaerobic conditions using an activated gas generating kit (5% H_2_, 5% CO_2_, 5% O_2_, and 85% N_2_). The cultures were maintained for 96 h and observed daily. The incubated broth was streaked by plating on each of (1) Columbia agar (TM MEDIA, TMH 116, India) supplemented with 5% defibrinated sheep blood and *Helicobacter* selective supplement (Skirrow’s medium) (HIMEDIA, code FD 008, India) and (2) Columbia agar supplemented with 5% defibrinated sheep blood and *H. pylori* selective supplement (DENT). The Columbia agar media were also supplemented with Vitamino Growth Supplement (HiMedia, code FD025, India) according to the manufacturer’s instructions. The streaked plates were incubated at 37°C for 3-5 days under microaerobic conditions, and any suspected *Helicobacter* colonies were examined for colonial morphology. The plates were not discarded until 12 days.

### DNA extraction from fecal samples

Bacterial DNA was extracted from the fecal samples using the QIAamp DNA Stool Mini Kit (Qiagen, GERMANY) according to the manufacturer’s instructions.

### PCR assays of *Helicobacter* species

PCR assays were performed using the EmeraldAmp GT PCR Master Mix Kit (Takara Bio., Japan). The oligonucleotide sequences (Metabion, Germany) of the primers targeting the 16S rRNA of *Helicobacter* species were as follows: Forward primer 5′- AAC GAT GAA GCT TCT AGC TTG CTA-3 ′ and reverse primer 5′-GTG CTT ATT CGT GAG ATA CCG TCA T-3′ for the amplification of 398 bp as described previously [10]. Each 25 μL of the PCR mixture consisted of 12.5 μL of 2× master mix, 1 μL of each primer (20 pmol), 5 μL of DNA template, and 5.5 μL PCR-grade water. The cycling conditions consisted of one cycle of initial denaturation at 94°C for 5 min, followed by 35 cycles of denaturation at 94°C for 30 s, annealing at 54°C for 40 s, and extension at 72°C for 30 s. The final extension consisted of one cycle at 72°C for 7 min. The PCR products were electrophoresed after loading onto 1% agarose gel using a final concentration of 0.1-0.5 μg/mL ethidium bromide solution. The DNA molecular weight marker was used in the gel electrophoresis using a 100 bp ladder (Gel Pilot QIAGEN, USA). The gel was photographed by a gel documentation system (Alpha Innotech, CA, USA), and the data were analyzed using computer software.

## Results

### Bacteriological isolation of *Helicobacter* species in the fecal samples of camels

The results showed that *H. pylori* and non-*H. pylori* species could not be isolated from all fecal samples using selective media.

### Prevalence of *Helicobacter* spp. in the fecal samples of camels by PCR

**Figure-1:**
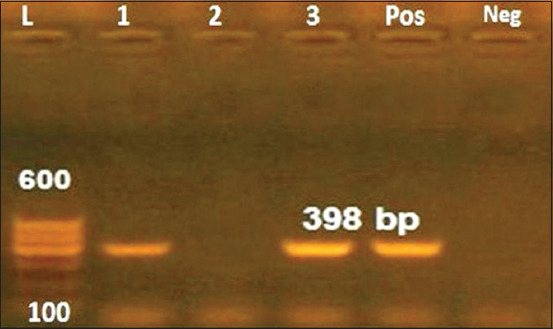
Agarose gel electrophoresis of polymerase chain reaction performed on *Helicobacter* spp. strains for the detection of 16S rRNA gene amplification product with a size of 398 bp. Lanes 1, 3 were positive; lane 2 was negative; L indicated the DNA ladder (100 bp ~ 600 bp) and Pos showed the control positive (*H. pylori* DNA) and Neg showed the control negative.

*Helicobacter* DNA was detected in the fecal samples of camels from 2 (22.22%) of the 9 camel farms. Of the 32 examined fecal samples of camels, 4 (12.5%) were positive for *Helicobacter* species. The farms that showed positivity included one farm from Ismailia center in which 1 (50%) of 2 camels’ samples was positive and another farm located in Qantarah center in which 3 (75%) of 4 camels’ samples were positive for *Helicobacter* species (Table-1 and Figure-1).

**Table-1:** The prevalence of *Helicobacter* spp. infection among examined camels.

No. of farms	Number of camels examined	Number of positive camels/(%)	Farms locations	Positive farms/(%)
1	3	0	Ismailia	Negative
2	2	1 (50%)	Ismailia	Positive
3	7	0	Ismailia	Negative
4	5	0	Qantarah	Negative
5	4	3 (75%)	Qantarah	Positive
6	2	0	Qantarah	Negative
7	5	0	Qantarah	Negative
8	3	0	Al Tal El Kabir	Negative
9	1	0	Fayid	Negative
Total =9	32	4 (12.5%)		2 (22.22%)

## Discussion

Non-*H. pylori* species can infect both humans and animals, and their probable transmission between humans and animals might serve as a reservoir for the transmission of pathogenic microorganisms to human contacts [11]. *In vitro* cultivation of the gastric non-*H. pylori* species organisms is extremely difficult and cannot be exclusively used for diagnostic purposes, which necessitates different techniques for the diagnosis of *Helicobacter* infections [7-12]. In the present study, using PCR, we identified the presence of *Helicobacter* spp. in the fecal samples of camels. The failure of isolation of *Helicobacter* species in camels that were detected by PCR indicated that *Helicobacter* infections in camels were associated with infection by non-*H. pylori* species other than the culturable species. Furthermore, the detection of *Helicobacter* spp. DNA in two camel farms from different localities indicated the spread of infection among the camels.

It has been reported that humans can be infected by different *Helicobacter* spp., the majority of which are common or potentially pathogenic, and most of which are probably zoonotic infections transmitted due to contact with animals [4]. Contact with cats, dogs, cattle, and pigs has been associated with *Helicobacter* infections in humans [4,13]. The camels examined in this study were reared in households in close contact with humans and other animals. Therefore, there is a possibility that camels could be one of the reservoirs of human infections by *Helicobacter* species. Several previous studies have supported the possibility of zoonotic infections of *Helicobacter* species. In the previous studies, *H. pylori* was isolated from sheep milk, based on which its zoonotic transmission from sheep was proposed [5,14]. Flahou *et al*. [15] found that *Helicobacter suis* colonized the stomach of asymptomatic rhesus and cynomolgus monkeys and suggested that *H. suis* infections in pigs possibly originated from nonhuman primates as a host jump from macaques to pigs that occurred thousands of years ago and that pig domestication has had a significant impact on the spread of *H. suis* in the pig population, from where this pathogen occasionally infects humans.

Our results supported by previous studies for detection of *H. pylori* DNA in camel’s milk in Iran by prevalence of (23.4%) [16], (13.33%) [17] and (5%) [18]. However, these studies focused on the detection of *H. pylori* species in milk of camels, whereas our study included the detection of *Helicobacter* spp. in the feces of camels.

## Conclusion

To the best of our knowledge, this is the first report of the detection of *Helicobacter* species infections in *C. dromedarius*. Further research is needed to clarify *Helicobacter* species infections in camels in terms of the epidemiological features and genome characterization.

## Authors’ Contributions

AY, AA and AH: Designed the study, collected the data, performed the laboratory work (PCR) and drafted the manuscript. AY and ME: Shared in the conception of the research idea, planned laboratory work, helped in the manuscript preparation. All authors discussed the results and commented on the manuscript and contributed to the final version of the manuscript. All authors have read and approved the final manuscript.
